# Cell Cycle Regulation of DNA Polymerase Beta in Rotenone-Based Parkinson's Disease Models

**DOI:** 10.1371/journal.pone.0109697

**Published:** 2014-10-10

**Authors:** Hongcai Wang, Yan Chen, Jinbo Chen, Zhentao Zhang, Wansheng Lao, Xizhi Li, Jinsha Huang, Tao Wang

**Affiliations:** 1 Department of Neurology, Affiliated Hospital of Binzhou Medical University, Binzhou City, Shandong Province, China; 2 Department of Gastroenterology, Union Hospital, Tongji Medical College, Huazhong University of Science and Technology, Wuhan, China; 3 Department of Neurology, Union Hospital, Tongji Medical College, Huazhong University of Science and Technology, Wuhan, China; 4 Department of Neurology, Renmin Hospital of Wuhan University, Wuhan, China; 5 Department of Surgery, Yangxin traditional Chinese medical hospital, Yangxin County, Binzhou City, Shandong Province, China; Hospital General Dr. Manuel Gea González, Mexico

## Abstract

In Parkinson's disease (PD), neuronal cells undergo mitotic catastrophe and endoreduplication prior to cell death; however, the regulatory mechanisms remain to be defined. In this study, we investigated cell cycle regulation of DNA polymerase β (poly β) in rotenone-based dopaminergic cellular and animal models. Incubation with a low concentration (0.25 µM) of rotenone for 1.5 to 7 days resulted in a flattened cell body and decreased DNA replication during S phase, whereas a high concentration (2 µM) of rotenone exposure resulted in enlarged, multi-nucleated cells and converted the mitotic cycle into endoreduplication. Consistently, DNA poly β, which is mainly involved in DNA repair synthesis, was upregulated to a high level following exposure to 2 µM rotenone. The abrogation of DNA poly β by siRNA transfection or dideoxycytidine (DDC) treatment attenuated the rotenone-induced endoreduplication. The cell cycle was reactivated in cyclin D-expressing dopaminergic neurons from the substantia nigra (SN) of rats following stereotactic (ST) infusion of rotenone. Increased DNA poly β expression was observed in the substantia nigra pars compacta (SNc) and the substantia nigra pars reticulate (SNr) of rotenone-treated rats. Collectively, in the *in vitro* model of rotenone-induced mitotic catastrophe, the overexpression of DNA poly β promotes endoreduplication; in the *in vivo* model, the upregulation of DNA poly β and cell cycle reentry were also observed in the adult rat substantia nigra. Therefore, the cell cycle regulation of DNA poly β may be involved in the pathological processes of PD, which results in the induction of endoreduplication.

## Introduction

Neuronal cells are maintained in the G2 phase for a long time, and endoreduplication has been verified as a pathological event in Parkinson's disease (PD) that occurs in cells prior to apoptosis [1]. Binucleated neurons have been shown to be present in the hippocampus of Alzheimer's disease (AD) patients [2]. Although the underlying mechanisms of cell cycle reentry and mitotic catastrophe are not well understood, the maintenance of the G2 state is suspected to be associated with endoreduplication [3], which is considered to be the default program for the canonical cell cycle during mitotic catastrophe [4]. In general, the regulation of endoreduplication in the mitotic cell follows the fundamental principles of cell cycle control and DNA replication [5]. However, the molecular control mechanisms of endoreduplication during neurodegeneration remain unclear.

Endoreduplication is a cell cycle variation that results in nuclear polyploidization by repeated rounds of DNA replication without cell division. Emerging evidence suggests that the correlation of endoreduplication with genome instability is reciprocal: endoreduplication leads to genome instability, and genome instability induces endoreduplication. However, the molecular mechanism by which genotoxic stress and cell cycle regulatory factors trigger endoreduplication is not clear [5]. Studies concerning DNA replication have helped increase the understanding of endoreduplication. In eukaryotic cells, DNA polymerases, which synthesize DNA by adding additional nucleotide triphosphates to an existing DNA molecule, are essential for DNA replication. DNA polymerase β (poly β), which is primarily involved in DNA repair, is also involved in DNA endoreduplication during normal development [6]. In neurodegenerative diseases, such as AD, the action of DNA poly β but not DNA α is loaded into DNA replication forks and results in aberrant DNA replication; erratic expression of DNA β occurs early in neuronal degeneration [7]; DNA poly β and the base excision repair pathway are required to repair the damage caused by oxidative stress [8]. Furthermore, evidence has indicated that the overexpression of DNA poly β enhances genome instability [9]. However, the connection between DNA poly β and endoreduplication in the neuronal cell cycle is unknown.

Rotenone, a mitochondrial complex I inhibitor, induces selective dopaminergic neuron damage [10] and endoreduplication [11]. In this study, to explore the regulatory mechanisms by which the G2 state is maintained prior to neuronal cell death in PD, we investigated the role of DNA poly β in rotenone-induced cellular and animal models.

## Materials and Methods

### Cell culture

Human neuroblastoma SH-SY5Y cells were acquired from the American Type Cell Culture Collection (ATCC, USA) and maintained in culture medium (DMEM-F12 ratio 1∶1, HyClone) containing 10% heat-inactivated fetal bovine serum (GIBCO-BRL Life Technology) in a 5% CO_2_ humidified incubator at 37°C.

### Reagents

Rotenone, DMSO, propidium iodide (PI), dideoxycytidine (DDC) and Hoechst 33258 were obtained from Sigma Chemical Co. Sheep anti-tyrosine hydroxylase (anti-TH) antibody (ab113), rabbit anti-DNA polymerase β antibody (ab175197) and mouse monoclonal anti-β-actin antibody (ab6276) were purchased from Abcam. Rabbit anti-cyclin D (#2978) and mouse anti-cyclin E (#4129) antibodies were purchased from Cell Signaling Technology and were used at a dilution of 1∶1000. Fluorophore-conjugated secondary antibodies were obtained from Invitrogen and used at a dilution of 1∶150–1∶250, and horseradish peroxidase (HRP)-conjugated antibodies for western blot detection were purchased from Cell Signaling Technology.

### Western blot analyses

Total protein lysates were isolated from the cells using RIPA lysis buffer (Pierce) supplemented with protease and phosphatase inhibitors. The protein concentration was determined using a BCA protein assay (Pierce). The cell lysates were separated using SDS-PAGE and electrophoretically blotted onto PVDF membranes (Millipore). The membranes were then probed with antibodies against DNA poly β, cyclin E or cyclin D overnight at 4°C. The signals were detected using KODAK PROFESSIONAL Film Developer. The protein levels were normalized against the levels of β-actin, and the optical density of each band was quantified.

### Hoechst staining

SH-SY5Y cells were grown on coverslips and exposed to rotenone. Differentiated SH-SY5Y cells were fixed with paraformaldehyde and permeabilized with 0.1% Triton X-100. Nuclear counterstaining was performed with 5 µg/mL Hoechst for 10 min. The cells were mounted onto slides using antifade mounting medium, and the coverslips were sealed with nail polish to prevent desiccation and the movement of the samples when observed under the microscope.

### BrdU/PI analysis

Cells (1×10^6^ cells/mL) were incubated with 10 µM BrdU (No. B5002, Sigma) for 30 min at 37°C in a controlled atmosphere. The cells were resuspended in 1 mL of 2 M HCl and incubated for 30 min at room temperature. The cells were then resuspended in 1 mL of Borax buffer (0.1 M sodium tetraborate-10-hydrate in H_2_O), followed by labeling with 5 µL of mouse purified anti-BrdU mAb (No. 317902, clone MoBU-1, Biolegend) for 1 h at 4°C in the dark. The cells were resuspended in 200 µL of wash buffer and labeled with 4 µL of goat-anti-mouse FITC-conjugated antibody for 30 min at 4°C in the dark. The cells were then resuspended in 200 µL of wash buffer, and 200 µL of PI buffer (3.4 mM trisodium citrate, 9.65 mM NaCl, 20 mg/mL PI and 0.03% Nonidet P-40 in H_2_O) was added to the cells, followed by incubation for 30 min at 4°C in the dark. The data analyses were performed using a flow cytometer equipped with a 488 nm argon laser and FlowJo 7.6.1 software.

### Small interfering (siRNA) construction

DNA poly β small interfering RNA (siRNA) and control siRNA were constructed by Ambion. They were dissolved in RNase-free water to prepare stock solutions of 20 µM, which were stored at −20°C. The following oligodeoxynucleotide (ODN) sequences were synthesized to target a specific sequence of human DNA poly β (TCAGCGAATTGGGCTGAAATA): antisense, 3′-dTdT AGUCGCUUAACCCGACUUUAU-5′ and sense, 5′-UCAGCGAAUUGGGCUGAAAUA dTdT-3′. SH-SY5Y cells were transfected with 50 nM DNA poly β siRNA or non-targeting control siRNA (nonspecific random sequence) using the Lipofectamine 2000 reagent (Invitrogen), and the cells were incubated for 48 to 96 h prior to treatment.

### Rotenone-based stereotactic (ST) infusion *in vivo* model of PD

First, 100 mg of rotenone was dissolved in 10 mL of dimethyl sulfoxide (DMSO) (final concentration, 10 µg/μL). Adult female Sprague-Dawley (SD) rats (230–280 g; Center of Experimental Animals, Tongji Medical College, Huazhong University of Science and Technology, China) were randomly divided into three groups: ST infusion of rotenone at 12 µg/1.2 µL into the right SNc (rotenone group, n = 15); ST infusion of 1.2 µL of solvent (containing 1.2 µL DMSO) (vehicle group, n = 15); and normal SD rats (normal group, n = 15). The infusion method and the evaluation of the rotenone-induced parkinsonian rodents are described in a previous publication [12]. All animals were acquired and cared for in accordance with the guidelines published in the NIH Guide for the Care and Use of Laboratory Animals (National Institutes of Health Publication No. 85–23, revised 1985). This study was approved and monitored by the Ethical Committee on Animal Experimentation of Tongji Medical College, Huazhong University of Science and Technology, China. The animals were sacrificed six weeks after the ST infusion of rotenone. Paraffin-embedded tissues were cut through the SNc (from −4.5 to −6.2 mm caudal to the bregma) using a sledge microtome (Thermo Scientific). The sections were de-waxed and rehydrated. Endogenous peroxidase activity was blocked by incubation in 0.3% hydrogen peroxide (H_2_O_2_) for 30 min. After antigen retrieval, the slides were incubated with 5% bovine serum albumin (BSA) for 60 min. The sections were then incubated with a TH antibody (1∶1000 dilution, Abcam), cyclin D antibody or DNA poly β antibody overnight at 4°C and were then incubated with fluorescein-conjugated secondary antibodies at 37°C for 1 h. The number of TH-positive cells was counted manually on bright-field microscopic images from three independent individuals. Cell counting was performed on the same coordinates of the brain section.

### Statistical Analysis

The data are expressed as the mean ± SD. Statistical comparisons of the results were performed using ANOVA. Significant differences (*P*<0.05) between the means of the control and treated cells were analyzed using t-tests.

## Results

### Alterations in neurites, axons and nuclear size

**Figure 1:**
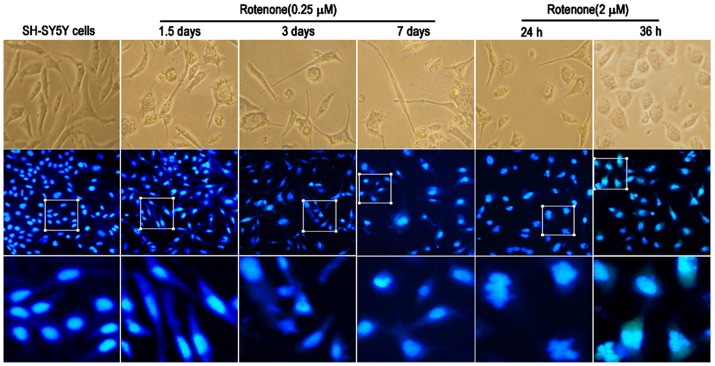
Alterations in cell morphology and nucleus size caused by treatment with rotenone (0.25 or 2 µM) (x200 and selective enlargement). Cellular morphological changes and axons alterations were visualized using a light microscope after exposure to rotenone (0.25 or 2 µM). The toxic effect of chronic exposure to rotenone at low-dose levels was maintained for 1.5, 3 and 7 days. With exposure to rotenone (2 µM), cells were cultured for 24 and 36 h. Under the same conditions, cells were stained with Hoechst dye and the nucleus size was observed using a fluorescence microscope. The selective enlargement graph was at the bottom.

Morphological features of control and treated cells were observed using phase-contrast microscopy. Compared to the control group, exposure to rotenone (0.25 or 2 µM) for different time periods induced apparent alterations in cell morphology. In the absence of rotenone, SH-SY5Y cells exhibited round, bright-phase cell bodies and long neurites. Exposure to 0.25 µM rotenone decreased the number of neurites and resulted in a flattened cell body, particularly after 7 days of exposure. When the cells were cultured with 2 µM rotenone, neurite outgrowth was severely impaired (Fig. 1).

The cells were fixed, and the nuclear size was observed following Hoechst staining. Although the cells occasionally showed binucleation or multi-nucleation after treatment with 0.25 µM rotenone, in most cases, the size of the nucleus was not altered from 1.5 to 7 days. In contrast, cells cultured with 2 µM rotenone for 24 and 36 h were binucleated or occasionally multi-nucleated. Simultaneously, nuclear shrinkage and karyorrhexis were present.

### Cell cycle redistribution

**Figure 2:**
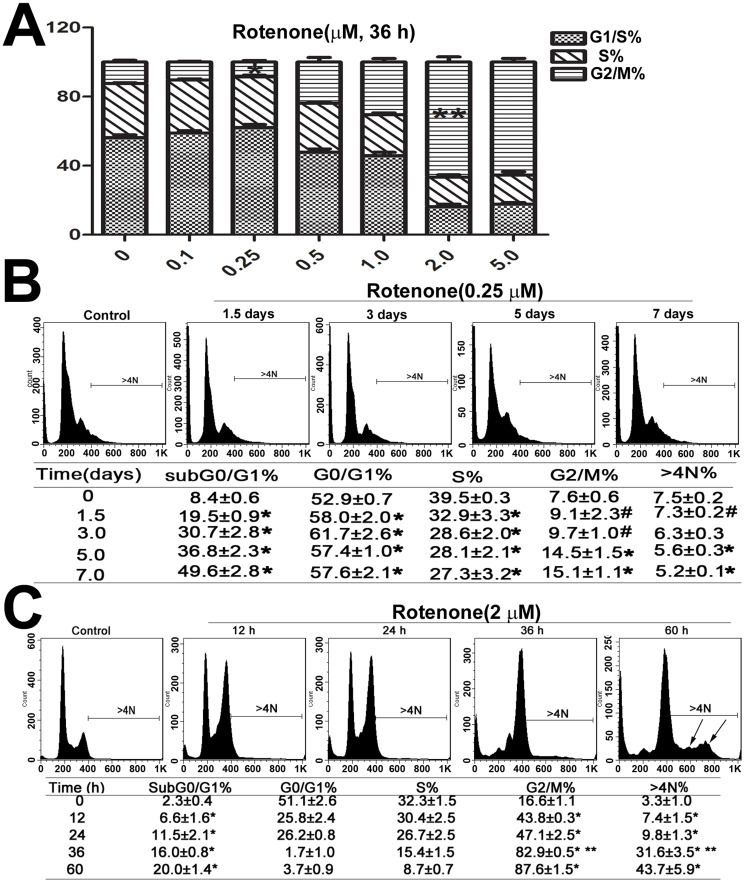
Modulation of cell cycle distribution after exposure to rotenone. Cell cycle analysis was performed using flow cytometer after propidium iodide (PI) staining of target cell DNA. The proportion of cells in each phase was statistically analyzed. The data were collected from three independent experiments and were expressed as the mean ± SD. (A) The cell cycle distribution was altered in response to different concentrations of rotenone for 36 h. * *P*<0.05 compared to the control group; ** *P*<0.05 compared to the 1 µM rotenone group. (B) and (C) Cells were cultured with 0.25 µM and 2 µM rotenone for different time periods. * *P*<0.05 compared to the control group; # *P*>0.05 compared to the control group; ** *P*<0.05 compared to the 24 h rotenone group; “>4N” represents cell DNA contents>4N.

The cells were exposed to different concentrations of rotenone, and exposure to 0.25 and 2 µM rotenone resulted in altered cell cycle distribution (Fig. 2 A). When the cells were treated with 0.25 µM rotenone, the proportion of cells in the sub G0/G1 phase increased in a time-dependent manner from 1.5 to 7 days. G0/G1 alterations were apparent after 1.5 days, although there was no significant difference in the number of cells in the G0/G1 phase from 1.5 to 7 days because of rotenone toxicity. Similarly, the number of cells in S phase was reduced from 1.5 to 7 days. The percentage of cells in G2/M phase increased after treatment for 5 and 7 days. Furthermore, the amount of cells with a DNA content>4N decreased after 5 days of rotenone treatment (Fig. 2 B).

In our experiments, rotenone (2 µM) induced an acute effect in the cultured cells. The cell cycle distribution was determined according to the DNA content of the cells, as evaluated by PI staining. When the SH-SY5Y cells were cultured with 2 µM rotenone, the percentage of cells in the sub G0/G1 phase (apoptotic cells) increased from 2.3±0.4% to 20.0β±1.4%. Moreover, the proportion of cells in G2/M continuously increased from 12 to 60 h, with obvious G2/M phase and with a DNA content>4N alterations from 24 to 36 h, which is consistent with increased endoreduplication. After 60 h, rotenone nearly induced a new cell cycle, as demonstrated by the DNA content>4N (Fig. 2 C).

**Figure 3:**
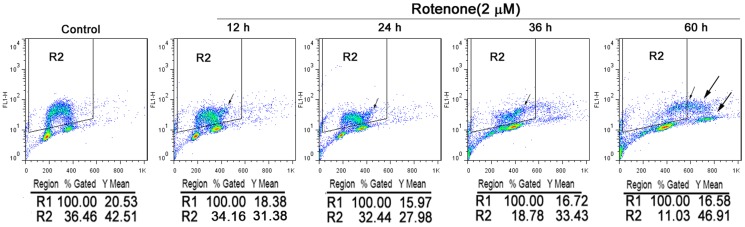
The cell DNA replication was examined by BrdU incorporation. The binding fluorescence intensity of FITC by flow cytometer determined the initiation of DNA replication. R2 represents cells are under replication. Small and big arrow represents that cells continues DNA replication with DNA contents of 4N.

The rotenone-induced alterations in DNA replication were evaluated using flow cytometry to quantify the number of cells actively incorporating BrdU. As shown in Fig. 3, the %Gate of the cells undergoing DNA replication continuously decreased over time after rotenone treatment. However, the arithmetic mean fluorescence (Y Mean), which represents the mean BrdU content, was elevated at 36 and 60 h. We also observed that the number of cells with a DNA content>4N, i.e., cells undergoing endoreduplication, increased from 12 to 60 h of rotenone treatment (small arrow, Fig. 3). Moreover, after 60 h of exposure to rotenone, a new cell cycle was induced, with similar proportions of cells in S phase and G phase (big arrow, Fig. 2 C and Fig. 3).

### Overexpression of DNA poly β promoted rotenone-induced endoreduplication

**Figure 4:**
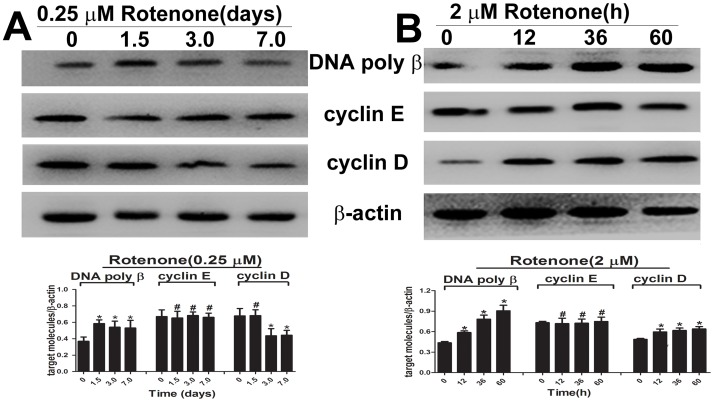
DNA poly β, cyclin E and cyclin D expression were detected after treatment with low and high concentrations of rotenone. The expression of DNA poly β, cyclin E and cyclin D were investigated by western blot. * *P*<0.05 compared to the control group; # *P*>0.05 compared to the control group.

**Figure 5:**
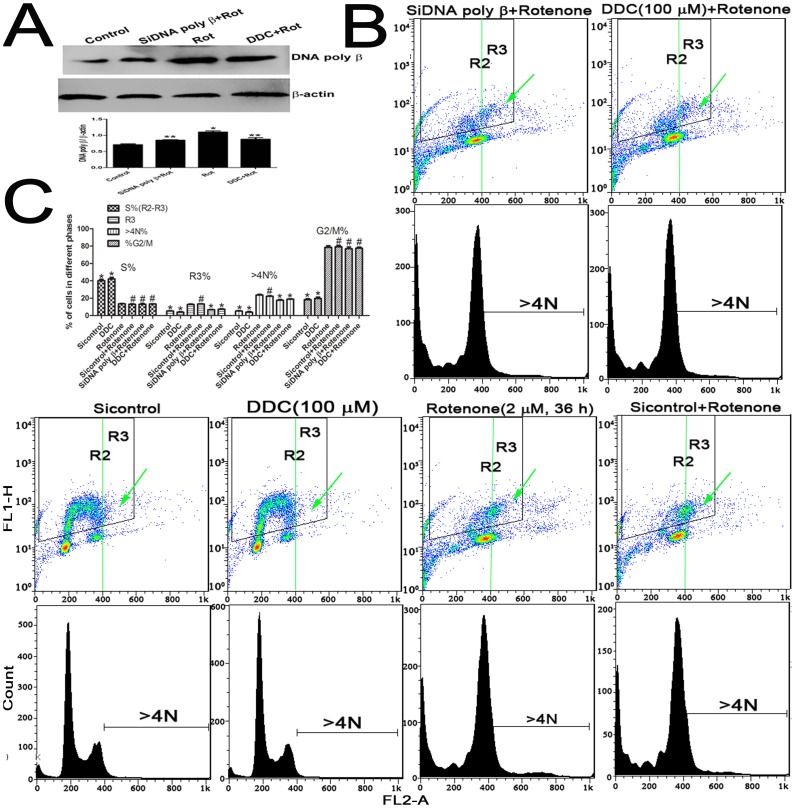
The cell cycle distribution in>4N phase and endoreduplication were interrupted by treatment with a DNA poly β inhibitor or by DNA poly β knockdown. (A) The expression of DNA poly β was examined by western blot as cells cultured with DDC or were transfected with siRNA DNA poly β. The results are presented as the ratio of the DNA poly β expression to β-actin expression. * *P*<0.05 compared to the control group; ** *P*<0.05 compared to the rotenone group; Rot: rotenone. (B) and (C) Cell cycle distribution and DNA replication were analyzed by flow cytometry using BrdU/PI staining after the addition of DDC or DNA poly β depletion. * *P*<0.05 compared to the rotenone group; # *P*>0.05 compared to the rotenone group; R3 represents endoreduplication; R2-R3 represents S phase; The data represent three independent experiments and are expressed as the mean ± SD.

When the cells were treated with 0.25 µM rotenone, DNA poly β expression increased and was maintained from 1.5 to 7 days. The key molecules for the G0/G1 and S phase transitions, cyclin D and cyclin E, were also detected. The expression of cyclin E was not altered, and cyclin D expression decreased after 3 days (Fig. 4 A). In cells cultured with 2 µM rotenone, the expression of DNA poly β was apparently elevated in a time-dependent manner from 12 to 60 h (Fig. 4 B). The expression level of cyclin E was not obviously altered, and cyclin D expression increased after 12 h. When the cells were cultured with the poly β inhibitor DDC and rotenone for 36 h, the expression of poly β was dramatically reduced. The SH-SY5Y cells were then transfected with a pool of siRNAs targeting DNA poly β, followed by exposure to 2 µM rotenone for 36 h; the expression of DNA poly β protein obviously decreased compared with that in the rotenone group (Fig. 5 A).

When endogenous DNA poly β was depleted via siRNA transfection and the cells were cultured with rotenone, the percentages of endoreduplicating cells (R3, green arrow) and cells with>4N DNA content were decreased compared with the percentages for mock-transfected cells (Fig. 5 B and C). The addition of the poly β inhibitor DDC to the rotenone-treated cells also led to a decrease in the percentages of endoreduplicating cells (R3) and cells with>4N DNA content. Moreover, the rotenone-induced alterations of cells in S phase (R2-R3) and G2/M phase were not affected by the addition of siRNA or DDC. However, in the mock-transfected cells, the rotenone-induced cell cycle alterations were not altered (*P*>0.05).

### Increased expression of DNA poly β during the rotenone-induced death of SNc neurons

**Figure 6:**
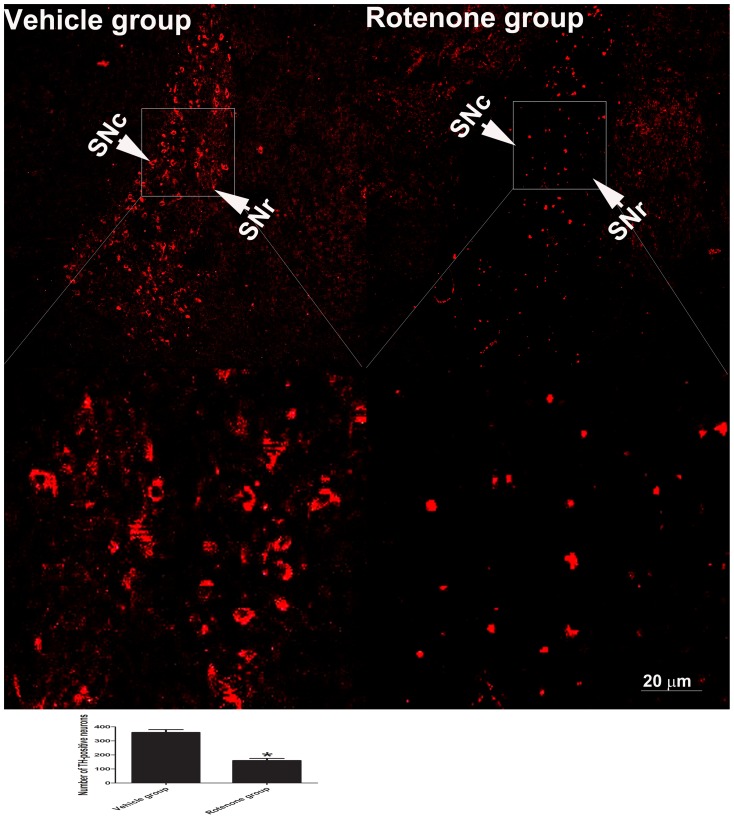
Expression of TH in the lesioned SN. Brain tissue sections were cut to a thickness of 5 µm and were immunostained using an anti-TH antibody. The TH-positive neurons were visualized using a Cy3-conjugated donkey-anti-sheep IgG antibody. The TH-positive SN neurons were counted in the same coordinates of two coronal sections from the right brain of two rats. The bottom graph is the selective enlargement. * *P*<0.05 compared to the vehicle group; SNc: substantia nigra pars compacta; SNr: substantia nigra pars reticulate. Scale bar = 20 µm.

Immunohistostaining of rat brain tissues using a TH antibody identified TH-positive neurons in the substantia nigra of rats in the rotenone, vehicle and normal groups. Vehicle infusion did not decrease the number of TH-positive neurons compared with the number in the normal group (data not shown); however, a loss of TH-positive neurons was observed unilaterally in the same coordinates of the substantia nigra of rotenone-treated rats relative to the vehicle group (Fig. 6). Rotenone also induced the accumulation of TH in neuronal cytoplasm in the substantia nigra of rotenone-treated rats.

**Figure 7:**
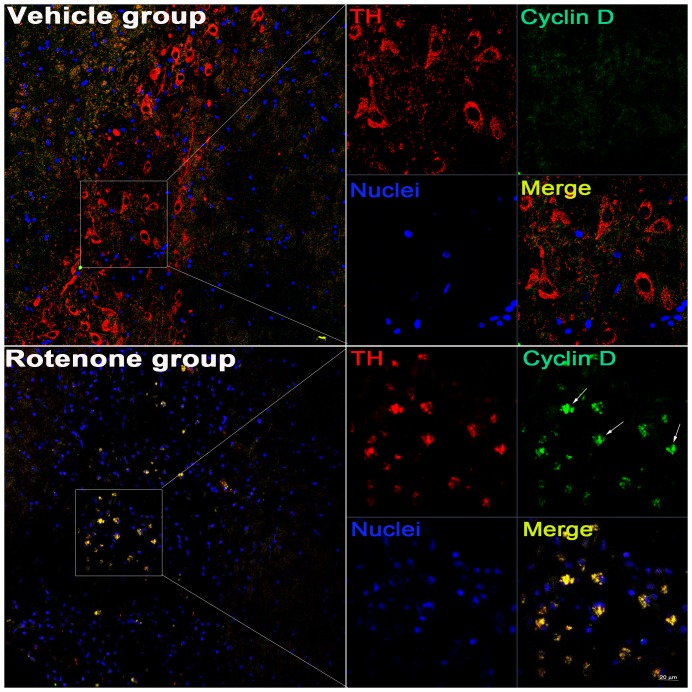
After stereotactic (ST) infusion rotenone, the increased expression of cyclin D was induced in the SN neurons. The immunoreactivity of cyclin D and TH was observed under the Laser confocal microscopy using DyLight 488-conjugated donkey-anti-rabbit IgG and Cy3-conjugated donkey-anti-sheep IgG, respectively, in the SN dopaminergic neurons of rats; The right graph is the selective enlargement; Scale bar = 20 µm.

**Figure 8:**
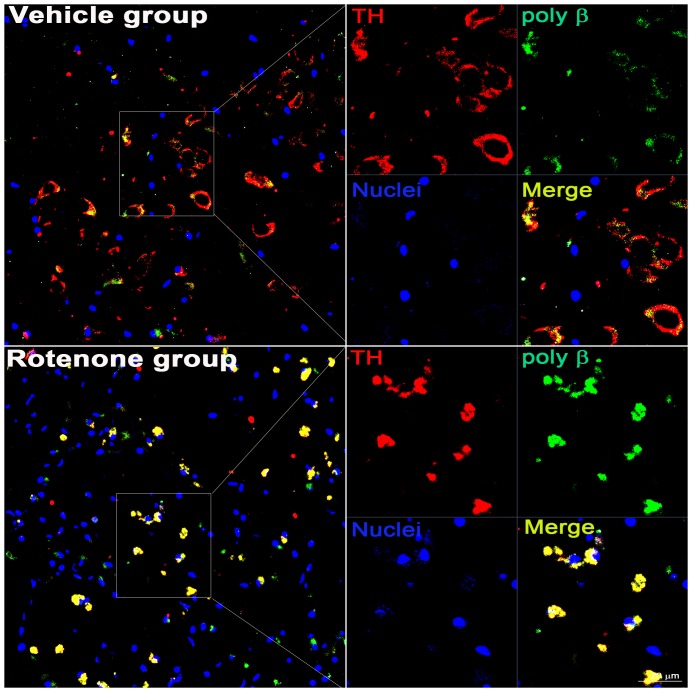
The expression of DNA poly β was enhanced in the lesioned brains of rats following rotenone infusion. Double immunohistostaining of DNA poly β and TH was performed. The right part represents the selective enlargement. Scale bar = 20 µm.

**Figure 9:**
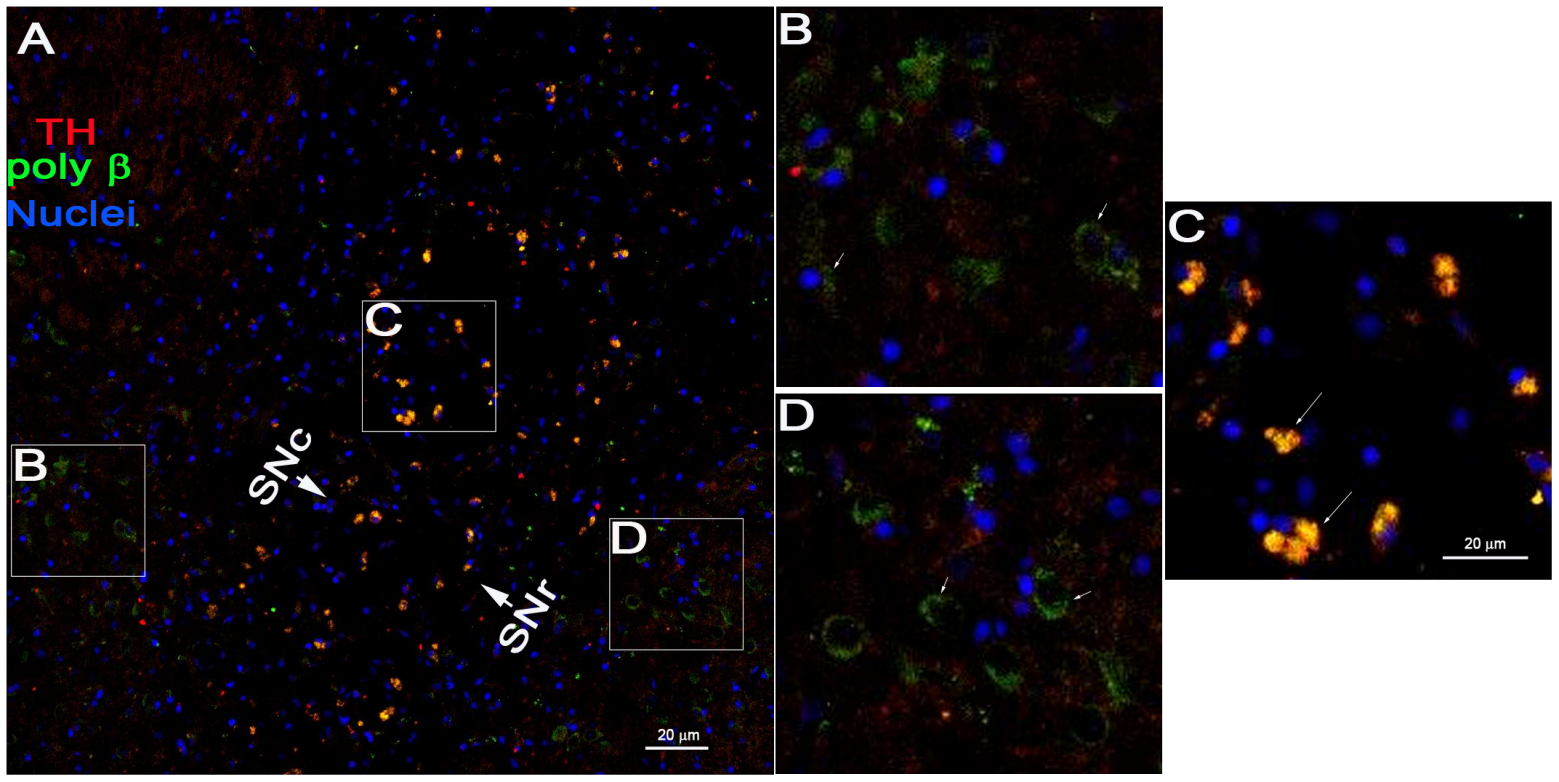
The selective increase of DNA poly β in the SN neurons in the ST-infused rats. After rotenone infusion, the immunoreactivity of DNA poly β was detected. B-D show selective enlargements of A. Scale bar = 20 µm.

Immunofluorescent double labeling of cyclin D and TH indicated that cyclin D was upregulated in the TH-positive neurons of the substantia nigra pars compacta (SNc) and the substantia nigra pars reticulate (SNr) in the lesioned brain (arrow, Fig. 7). The morphological features of the dopaminergic neurons were not obviously altered after vehicle infusion (containing 1.2 µL DMSO) (Fig. 7). In the lesioned SNc and SNr, the immunoreactivity of DNA poly β was obviously increased in the TH-positive neurons (Fig. 8). Furthermore, in the rotenone-treated rats, a selective increase in DNA poly β and morphological alterations were observed in the dopaminergic neurons (long arrow, Fig. 9 C) but not in the cells outside of the substantia nigra (short arrow, Fig. 9 B and D).

## Discussion

In the present study, we demonstrated that the overexpression of DNA poly β was involved in the rotenone-mediated pathology of cellular and animal models of PD. In the cell culture model, increased levels of DNA poly β promoted rotenone-mediated endoreduplication. Selective injury to dopaminergic neurons by rotenone resulted in the upregulation of DNA poly β as the neuronal cell cycle was reactivated.

Mitotic catastrophe occurs during the apoptotic death of dopaminergic neurons [1], but the molecular mechanism by which the G2 state is maintained remains unclear. The induction of endoreduplication is associated with a delayed G2 phase prior to cell death in degenerative diseases [3]. In general, endoreduplication occurs in response to physiological stress [13] and to genotoxic stress [5]. In the rotenone-mediated dopaminergic cell model, we demonstrated that high doses of rotenone increased endoreduplication and G2/M arrest over time. A low concentration of rotenone blocked cell cycle progression, mainly at the G1/S phase, because of the decrease in endoreduplication. The alterations in the cell cycle distribution were also consistent with the observed morphological changes.

Under normal circumstances, DNA poly β is involved in the base excision repair pathway, which helps maintain genome stability [9]. DNA poly β is also involved in DNA endoreduplication during normal development [6]. Interestingly, evidence has indicated that the overexpression of DNA poly β enhances genome instability [9]. In neurodegenerative diseases, such as AD, DNA poly β is loaded onto the chromatin, resulting in aberrant DNA replication, and this process is independent of the base excision repair pathway [7]. In our rotenone-administered cellular models, the expression of DNA poly β was markedly elevated, and this overexpression promoted endoreduplication and genome instability. Generally, DNA poly β is an essential component of the base excision repair pathway of DNA damage repair [8]. In degenerative dopaminergic neurons, the immunoactivity of DNA poly β was also detected in the lesioned substantia nigra, which suggests that DNA poly β might be involved in the pathological processes of PD.

Cyclin D is the key regulator of the mid-G1 transition, and increased levels of cyclin D can lead to neuronal re-entry into the cell cycle [14]. Cyclin D was found to be upregulated in primary cultures of embryonic rat midbrain neurons after exposure to 1-methyl-4-phenylpyridinium (MPP^+^) [1]. In our rotenone-administered rats, dopaminergic neurons from the SNc and SNr were intensely immunoreactive for cyclin D. We also observed an increase in cyclin D expression in the rotenone-cultured cells. While the cell cycle reentry in adult dopaminergic neurons, the immunoreactivity of DNA poly β was obviously enhanced in the lesioned SN. We also observed a selective increase in DNA poly β and the death of dopaminergic neurons in the SN upon rotenone infusion, which were consistent with the findings of a previous study [15]. In the lesioned SN, we also observed that the accumulation of TH in the neuronal cytoplasm was induced, which was similar with the previous findings that the pharmacological stimulation caused the altered subcellular localization of TH [16].

In summary, the overexpression of DNA poly β promotes rotenone-induced endoreduplication, which is associated with the maintenance of the G2 state. DNA poly β is overexpressed during cell cycle re-entry-associated dopaminergic neuronal death in rotenone-based animal models. Thus, in dopaminergic cells, rotenone-induced overexpression of DNA poly β enhances genome instability through the induction of endoreduplication; increased DNA poly β and cell cycle re-entry may be implicated in the loss of dopaminergic neurons. In the *in vivo* model, the correlation between DNA poly β and endoreduplication requires further investigation.
